# Cell Hierarchy and Lineage Commitment in the Bovine Mammary Gland

**DOI:** 10.1371/journal.pone.0030113

**Published:** 2012-01-13

**Authors:** Gat Rauner, Itamar Barash

**Affiliations:** 1 Institute of Animal Science, ARO, The Volcani Center, Bet-Dagan, Israel; 2 The Robert H. Smith Faculty of Agriculture, Food and Environment, The Hebrew University of Jerusalem, Jerusalem, Israel; Universitätsklinikum Carl Gustav Carus an der Technischen Universität Dresden, Germany

## Abstract

The bovine mammary gland is a favorable organ for studying mammary cell hierarchy due to its robust milk-production capabilities that reflect the adaptation of its cell populations to extensive expansion and differentiation. It also shares basic characteristics with the human breast, and identification of its cell composition may broaden our understanding of the diversity in cell hierarchy among mammals. Here, Lin^−^ epithelial cells were sorted according to expression of CD24 and CD49f into four populations: CD24^med^CD49f^pos^ (putative stem cells, puStm), CD24^neg^CD49f^pos^ (Basal), CD24^high^CD49f^neg^ (putative progenitors, puPgt) and CD24^med^CD49f^neg^ (luminal, Lum). These populations maintained differential gene expression of lineage markers and markers of stem cells and luminal progenitors. Of note was the high expression of Stat5a in the puPgt cells, and of Notch1, Delta1, Jagged1 and Hey1 in the puStm and Basal populations. Cultured puStm and Basal cells formed lineage-restricted basal or luminal clones and after re-sorting, colonies that preserved a duct-like alignment of epithelial layers. In contrast, puPgt and Lum cells generated only luminal clones and unorganized colonies. Under non-adherent culture conditions, the puPgt and puStm populations generated significantly more floating colonies. The increase in cell number during culture provides a measure of propagation potential, which was highest for the puStm cells. Taken together, these analyses position puStm cells at the top of the cell hierarchy and denote the presence of both bi-potent and luminally restricted progenitors. In addition, a population of differentiated luminal cells was marked. Finally, combining ALDH activity with cell-surface marker analyses defined a small subpopulation that is potentially stem cell- enriched.

## Introduction

The role of somatic stem cells and their progenitors in mammary gland development and renewal has been extensively studied in the human breast and in the mouse model. Manifestation of the cancer stem cell hypothesis, which identifies normal mammary stem cells (MaSC) and their immediate progenitors as putative targets for cell transformation and tumor initiation (reviewed in [1]), has further heightened interest in normal MaSC properties and regulation. In contrast, limited information is available on stem cells and their progeny in the mammary glands of other species. Thus, the aim of this study was to characterize the cell hierarchy and properties of distinct epithelial cell populations in the bovine mammary gland.

The presence of MaSCs with the capacity for multipotent differentiation in the mammary gland was depicted in early studies demonstrating the development of transplanted mammary fragments or epithelial cells into a rudimentary multilayered ductal network, composed of a luminal epithelial layer lined by contractile myoepithelial cells that are juxtaposed to the extracellular matrix and fatty stroma [2], [3], [4], [5], [6]. The putative stem cells were distinguished according to their orientation in the human breast [7] or their morphological properties—small round shape, pale staining and large spherical nuclei—in mice [5], [8]. Similar to other somatic tissues, a side population was identified in the mammary gland that exhibited Hoechst dye-effluxing [9], [10], [11]. Label retention was also associated with stemness [9], [12], [13], [14], [15], [16], [17]. Prospective isolation of mouse and human MaSC-enriched populations was achieved by fluorescence-activated cell sorting (FACS) according to the expression or activity of putative stem cell markers (reviewed in [6], [18]). Multipotency and self-renewal were confirmed for these cells by transplantation into the cleared mammary fat pad of a female mouse that was conditioned to support the propagation of human cells by pre- and co-transplantation of fibroblasts [19], [20]. Ultimately, single mouse mammary epithelial stem cells, isolated according to expression of the cell-surface markers CD24 and CD49f or CD29, were shown capable of reconstituting a functional mammary gland upon transplantation at limiting dilutions [21], [22].

*In-vitro* tests for stemness and progenitor activity in the human breast and mouse mammary gland were also developed [21], [22], [23], [24], [25]. The mammosphere assay for stemness is based on the ability of stem cells to escape anoikis and form floating spheres under conditions that do not permit adherence. The clonal assays monitor progenitor number and properties [26]. Together, these assays paved the way to dissecting signal-transduction pathways in stem cells and their progenitors which led, for example, to a better understanding of the role of Notch in normal mammary gland development and tumorigenesis [27], [28], the effect of the EGF/AKT pathway in initiating breast cancer [29], and the tumorigenic transformation process that generates breast cancer [30].

Distinguishing mouse MaSCs and their progenitors enabled tracking their numbers and dynamics during puberty, pregnancy and involution [31], [32], [33], [34], [35], [36], [37] and reviewed in [38]. This prompted insights into the role of MaSCs in mammary development, suggesting, for instance, that distinct subsets of MaSCs account for pubertal mammary development and its growth during pregnancy, and even for differences in resistance to tumor formation. Delineation of mammary epithelial cell hierarchy, as perceived today, consists of MaSCs giving rise to uni-potent luminal-restricted progenitors that, in turn, differentiate into alveolar myoepithelial or secretory cells. The MaSCs also generate bi-potent progenitors, which give rise to ductal epithelial or ductal myoepithelial cells [18].

Pioneering studies distinguished bovine mammary epithelial cells (bMECs) according to their morphology and DNA label retention [39], [40], [41], [42]. Sorting of bMECs according their ALDH enzymatic activity has also been recently reported, allowing the separation of luminal and basal compartments [43]. The latter compartment was enriched in stem cell-like activity, as confirmed by the development of spherical structures with limited projections after cell transplantation under the mouse kidney capsule. Indeed, xenotransplantation of bMECs into the cleared mouse mammary fat pad is extremely challenging due to putative inherent differences between the fatty mouse adipose stroma and the human or bovine fibrous ones [44], [45], [46]. In addition, an attempt to identify bovine mammary stem and progenitor cells by cell sorting according to surface-marker expression has never been reported. The limited progress in bovine MaSC (bMaSC) research compared to that in mice and humans has prevented comparable delineation of factors and signal-transduction pathways that affect these cells' self-renewal and differentiation processes, as well as dissection of the resulting cell hierarchy. Given that MaSCs are essential for mammary tissue regeneration with each cycle of lactation, isolation and characterization of bMaSCs and their progenitors is of primary interest not only to extend our knowledge of the diverse regulation of MaSCs among mammals, but also for the milk-production industry, as their activity may directly affect lactation persistency [47], [48]. Importantly, the literature is conspicuously lacking in reports of mammary tumors in cattle, raising the possibility that bMaSCs and their progenitors are potentially immune to malignant transformation, either as a property of the cells themselves or due to environmental or systemic cues. The structure of the bovine gland resembles that of the human breast as both share the milk-secreting unit termed terminal ductal-lobular unit (TDLU, [45], [46]). TDLUs are also the sites of neoplastic initiation and do not develop in the mouse gland, which contains the equivalent lobulo-alveolar(LA) unit [49], [50]. Thus, in addition to its productive properties, the bovine mammary gland and its cell populations may also serve as a unique model for studying tumor resistance and for developing new strategies against breast cancer development.

In the current study, isolated Lin^−^ epithelial cells were sorted into four distinct populations according to expression of the cell-surface markers CD24 and CD49f. Gene expression and immunohistochemical analyses supplemented by *in-vitro* tests established these populations as principal constituents of the bovine mammary gland cell hierarchy.

## Results

### Expression of lineage markers in the bovine mammary gland

**Figure 1:**
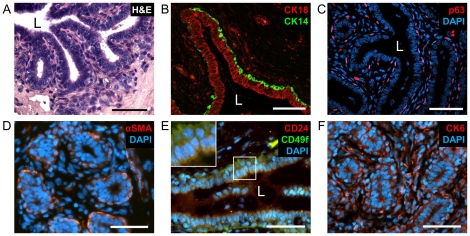
Luminal and basal/myoepithelial layers in the bovine mammary gland can be distinguished by immunofluorescence analysis. A: H&E staining of sections from a heifer's mammary gland reveals ductal structures penetrating the fibrous stroma. B–F: Immunofluorescence detection of mammary lineage markers. Inset: 2× magnification. L = lumen. Bar = 50 µm.

Two major cell lineages, luminal and basal/myoepithelial, stem from the asymmetrical division of a small population of cells with regenerative capacity and multipotency that resides in specific locations in the mammary gland. Cell hierarchy within these lineages has been extensively studied in the mouse mammary gland and the human breast. To examine the applicability of lineage markers established in these mammalian species for marking and separating the less investigated bovine mammary cell populations, their *in-situ* localization in the heifer's mammary gland was examined (Figure 1). Hematoxylin & Eosin (H&E)-stained cross and longitudinal paraffin sections of the heifer's mammary gland revealed ducts penetrating and branching within the fibrous stroma (Figure 1A). Immunostaining of potential lineage markers localized CK18 expression in the luminal compartment of the ducts, whereas CK14, p63 and αSMA expression was detected in the basal/myoepithelial layer (Figure 1B–D). These results confirmed previous findings for CK14, CK18 and αSMA localization [51], [52], and implicate p63 as a basal/myoepithelial marker in the bovine mammary gland. Expression of CD24 and CD49f was also examined *in situ*. Similar to the human breast and mouse mammary gland [22], [53], [54], [55], CD24 was detected in the luminal epithelium whereas CD49f was localized to the basal layer of the heifer's gland (Figure 1E). CK6 expression has been previously reported in the ductal luminal epithelium of the human breast and mouse mammary gland [24], [56], [57]. In contrast, it was primarily localized to the basal and stromal cells of the bovine mammary gland (Figure 1F). These findings suggest that CK6 is indeed useful for differentiating the basal/myoepithelial from luminal bMECs, but may not be suitable for distinction of basal from stromal cells. Taken together, the *in-situ* localization of these protein markers elaborates on the homology between bovine mammary gland and its murine and human counterparts. It also supports use of the mouse-based cell-separation system, which applies lineage markers CD24 and CD49f for prospective sorting and enrichment of the bMEC populations.

### Sorting primary bMEC suspension into four populations according to CD24 and CD49f expression in individual cells

**Figure 2:**
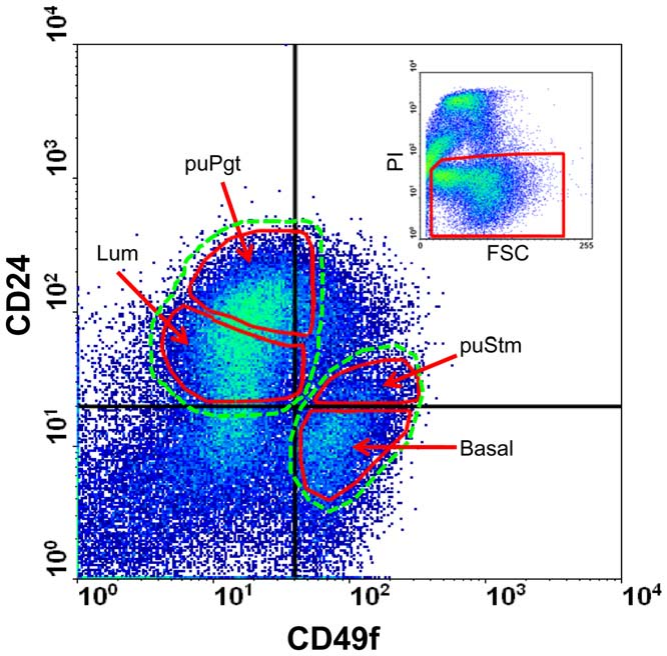
Four populations of epithelial cells with distinct CD24 and CD49f expression were identified in the bovine mammary gland. Lin^−^ bMECs from heifer mammary gland were sorted according to CD24 and CD49f expression. Two main populations: CD24^neg-med^CD49f^pos^ and CD24^med-high^CD49f^neg^, encircled by dashed green lines, emerged in the density plot. Putative populations enriched with stem cells (puStm, 5.8±1.3%) and their progenitors (puPgt, 8.0±1.4%), as well as their complementary Basal (11.7±2.9%) and luminal (Lum, 12.2±2.6%) populations (encircled by solid red lines) were collected. Inset: gating of living cells (framed in red) according to PI staining. Percentage of each population was calculated out of the total living cells detected. FSC – forward scatter.

bMECs were sorted into putative distinct populations according to the methodology used to distinguish mouse mammary epithelial cells adapted here to the specific characteristics of the bovine gland. Accordingly, mammary glands of 7- to 10-month-old heifers were dissociated into organoids, which were subsequently digested into a suspension of single cells, and Lin^−^ cells were then obtained using mouse antibodies to CD45, CD31and TER119. Propidium iodide (PI)-stained cells were gated by FACS before cell separation, and the relatively high amount of dead cells and cell debris (61±5% of events detected by FACS, Figure 2 inset) resulting from tissue digestion was excluded.

Two major epithelial cell populations emerged in the density plot analysis (Figure 2, dashed lines): CD24^neg-med^CD49f^pos^ and CD24^med-high^CD49f^neg^. A third population, CD24^neg^CD49f^neg^, represented non-epithelial cells. CD24 and CD49f are expressed in the luminal and basal/myoepithelial compartments, respectively. Thus, the CD24^med-high^CD49f^neg^ and the CD24^neg-med^CD49f^pos^ populations potentially represented the luminal and basal compartments, respectively. Further distinction was made within these populations, based on parameters used to enrich the mouse MEC suspensions for stem cells (CD24^med^CD49f^high^) and their progenitors (CD24^high^CD49f^low^) [21], [22]. Accordingly, their bovine counterparts, CD24^med^CD49f^pos^ and CD24^high^CD49f^neg^, were termed putative stem (puStm) and putative progenitor (puPgt), respectively (Figure 2, solid lines). For a comprehensive analysis of the poorly studied bMECs, the adjacent populations—CD24^neg^CD49f^pos^ and CD24^med^CD49f^neg^—were also collected and termed Basal and Luminal (or Lum), respectively. This nomenclature reflects relevant information from studies on the properties of mouse MEC populations [21], [22], [58], [59], [60]. To further characterize the separated populations, expression of genes encoding lineage protein markers was analyzed.

### Distinguishing the enriched bMEC populations by gene-expression analysis

bMECs from the mammary glands of four heifers were individually sorted into the four populations: puStm, Basal, puPgt and Lum. mRNA was extracted from cells of each population and the expression of genes, selected according to their involvement in marking and regulating breast/mammary gland cell hierarchy, was determined by RT-PCR.

#### Deciphering the basal/luminal origin of the sorted populations

**Figure 3:**
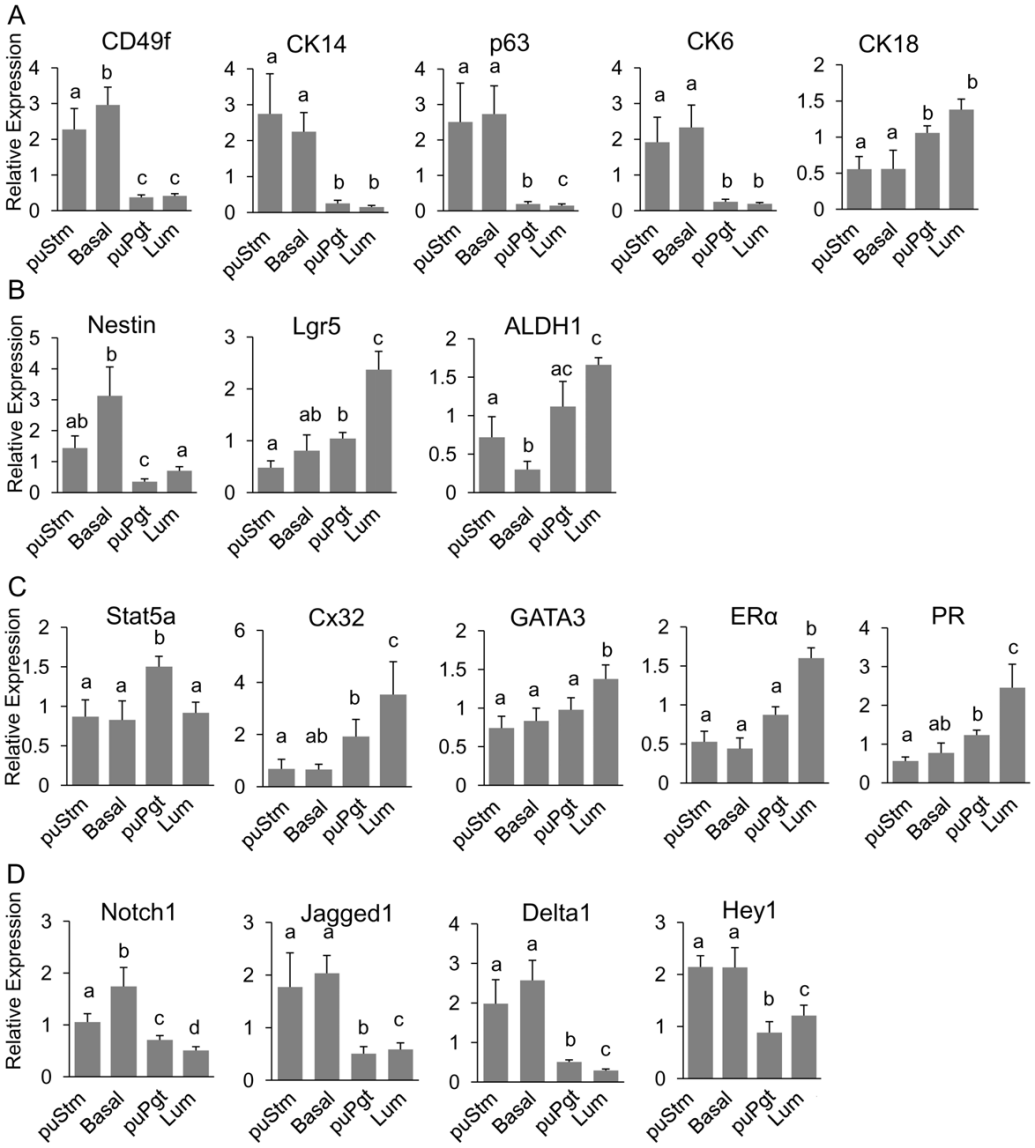
Delineation of bMEC populations by expression analysis. Expression levels of selected genes, relative to ungated Lin^−^ cells, were determined in the four sorted bMEC populations by real-time PCR. A: Differential mRNA expression of basal (CD49f, CK14, p63 and CK6) and luminal (CK18) markers infers the location of the sorted populations within the mammary tissue. B: Differential expression of genes implicated as stem and progenitor cell markers in several adult tissues. C: Differential expression of genes associated with luminal lineage. D: Differential expression of genes along the Notch pathway. Columns represent mean±SEM of data collected from four individual heifers and different letters above the columns indicate statistically significant (*P*<0.05) differences in the comparison of each value to its three counterpart values.

A clear distinction between the basal and luminal lineages, bordered by the dashed lines in Figure 2, was demonstrated (Figure 3A). CD49f, CK14, and CK6 were highly expressed in the puStm and Basal populations. In contrast, significantly (*P*<0.05) higher expression of CK18 marked the puPgt and Lum cells. This analysis supported the applicability of CD49f and its mouse-based detection system to separating the bovine cell population by FACS, and the unique expression of CK6 in the basal compartment of the bovine mammary gland.

#### Expression of putative stem cell markers

A main objective of separating bMECs is the prospective enrichment of stem cells. The expression of established markers, used to identify this rare population in various mammalian tissues, was therefore examined in the four bMEC populations (Figure 3B). Nestin is an intermediate filament protein regarded as a neural stem cell marker. Recently, it was also inferred as a putative stem cell marker in the human breast [61], [62], [63]. Among the bMEC populations, Nestin was highly expressed in the basal compared to the luminal compartment, but with no significant difference between the Basal and puStm cells. Nestin's lowest expression level in the puPgt cells distinguished them from the Lum population. Attempts to localize Nestin expression *in situ* failed due to the lack of bovine cross reaction of the bovine antigen with available antibodies (data not shown). Lgr5 is a G-protein-coupled receptor and a Wnt target gene, implicated as a stem cell marker in the intestinal and skin epithelium [64], [65], [66], [67]. Lgr5 has also been localized to a few, scattered epithelial cells in the basal layer of the adult mouse mammary gland [65]. In contrast, expression analysis of Lgr5 in the bMECs revealed a significant (*P*<0.05) two- to fourfold higher level of expression in the Lum cells compared to the other populations. This result might infer a unique role for Lgr5 in bovine mammary cell differentiation and highlights its possible use as a marker for the differentiated luminal bMEC population. The aldehyde-oxidizing enzyme ALDH1 was originally identified as a stem cell marker in the hematopoietic system [68] and later also in the human breast [43], [69]. A recent study, performed in bovine mammary cells, associated high ALDH activity with luminal cells, contrary to lower activity in myoepithelial progenitors [43]. Here, we found high expression of ALDH1in the luminal populations puPgt and Lum. However, its lower expression in puStm cells was still sufficiently high to distinguish this population from the Basal one that expressed ALDH1 at a threefold lower level.

#### Differential gene expression among the luminal bMEC populations

Studies in mouse and human mammary glands have demonstrated that the equivalents to the puPgt population (CD24^high^CD49f^neg^) are enriched with luminal-restricted progenitors [21], [22]. Cells composing the equivalent of the Lum fraction have been less investigated. Gene-expression analysis revealed significant differences between these two luminal populations in the bovine mammary gland (Figure 3C). Stat5a, a transcriptional regulator of lactation, controls the development of alveolar epithelium and luminal progenitors in the mouse mammary gland [70], [71], [72]. Its significantly higher (*P*<0.05) expression in the bovine mammary puPgt cells compared to the other cell populations supports the notion that CD24^high^CD49f^neg^ bMECs are enriched with luminal progenitors. Connexin 32 (Cx32), a protein in the gap junction complex, is located downstream of Stat5 signaling and is probably involved in mammary cell differentiation [71], [73]. As expected, Cx32 was expressed in the Lum population at twofold higher levels than in the puPgt cells and at 10-fold higher levels than in the puStm or Basal cell populations. GATA3 is a regulator of mammary luminal differentiation. Conditional suppression of its expression leads to expansion of the progenitor cell pool [60]. Thus, the significantly (*P*<0.05) higher levels of GATA3 expression in the Lum population relative to the other populations might mark cell differentiation within the luminal lineage. Importantly, the Lum population also maintained significantly higher mRNA levels of estrogen receptor α (ERα) and progesterone receptor (PR) compared to the other cell populations. Taken together, these results support characterization of the CD24^high^CD49f^neg^ (puPgt) cells as a progenitor-enriched population, and enrichment of the CD24^med^CD49f^neg^ (Lum) population with luminal-differentiating or mature cells.

#### Basally-located populations express higher levels of genes along the Notch1 pathway

Activation of the Notch pathway depends on concerted interactions between adjacent cells in the mammary epithelium, which express its different ligands, receptors and target genes. Thus, members of the Notch pathway were considered potential candidates to mark diversity among the bMEC populations. Gene-expression analysis (Figure 3D) revealed higher expression of all constituents of the Notch pathway in cells of the basal compartment (i.e. puStm and Basal) compared to those of the luminal one (puPgt and Lum). Interestingly, significantly higher expression of Notch1 receptor gene was measured in the Basal vs. puStm population, indicating potentially different properties of these two basally oriented populations. A difference in gene-expression pattern among the luminal populations was also noted: Notch1 and Delta1 were highly expressed in the puPgt vs. Lum cells, whereas Jagged1 and Hey1 were more highly expressed in the Lum-enriched cells.

### *In-situ* localization of selected proteins in the bovine mammary gland

**Figure 4:**
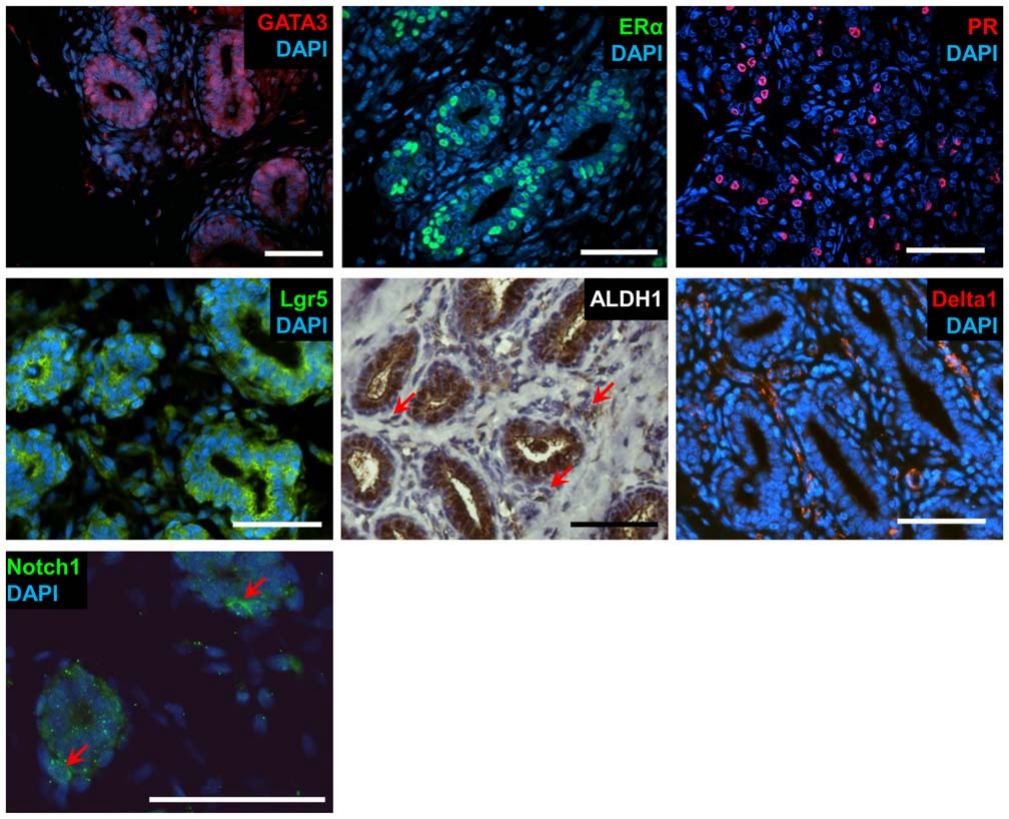
*In-situ* localization of proteins with distinct expression among the bMEC populations. All analyses depict immunofluorescence detection, except for ALDH1 which was detected by DAB reaction, generating a brown signal with hematoxylin counterstaining of the nuclei. ALDH1: red arrows mark positively stained cells in the stromal area. Notch1: red arrows mark positively stained cells in the basal layer. Bar = 50 µm.

Gene expression analysis in the enriched bMEC populations was complemented by *in-situ* localization of selected protein products in the distinct layers of the heifer's mammary gland (Figure 4). GATA3, ERα and PR were detected in the nuclei of some, but not all luminal cells of the bovine mammary ducts. Notably, these cells are typically in close contact with the basal layer and are not exposed to the lumen. Thus, the Lum subpopulation that expressed high levels of these genes may reside in a restricted niche within the bovine mammary duct. Lgr5 and ALDH1 were also detected in the luminal compartment, but their cytoplasmic staining did not allow further distinction of their localization. Nevertheless, individual stromal cells, probably endothelial cells and fibroblasts, also stained for ALDH1 (Figure 4, red arrows). In contrast to the above luminal-expressed proteins, Delta1 was mainly localized to the stromal cells of the bovine mammary gland and possibly also to the basal layer. Some of the positively stained stromal cells were likely endothelial cells and others, fibroblasts. Notch1 was detected in distinct, isolated cells of the basal layer and may mark a minor subpopulation of the bMEC population.

### Clonal assay and propagation rate of bMECs

**Figure 5:**
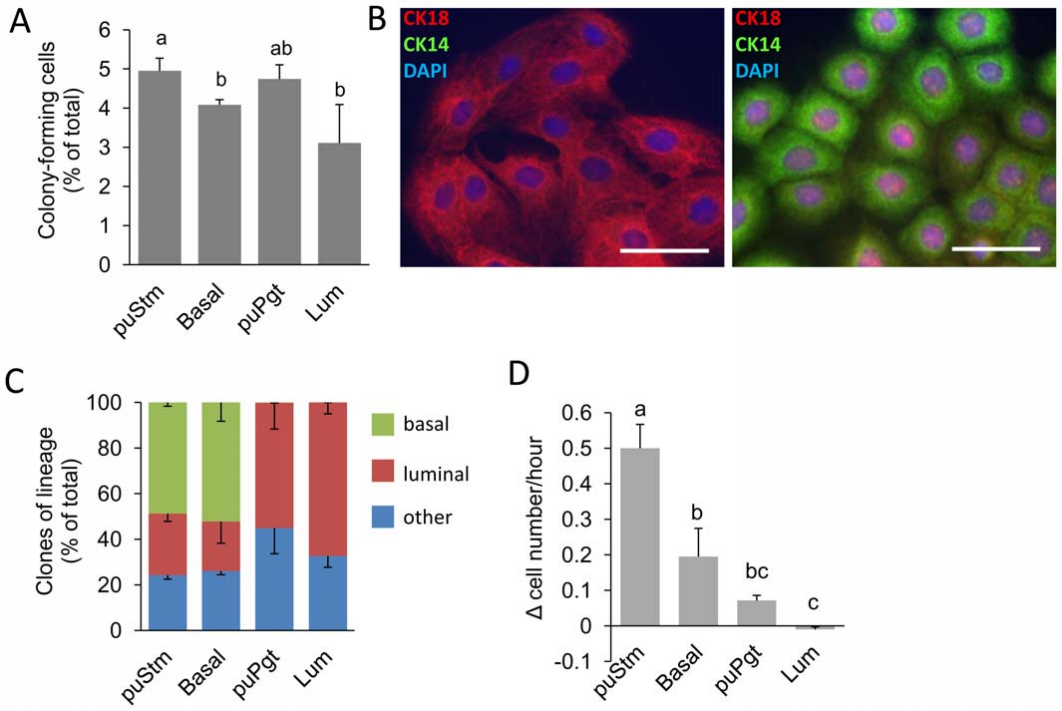
Multipotency, high propagation potential and clone-formation capability characterize the puStm population. A: Percentage of colony-forming cells out of the total sorted cell population. B: Immunofluorescence staining of adherent clones, demonstrating two types: luminal clones expressing CK18 (left) and basal clones expressing CK14 (right). C: Composition of clone types formed by each of the sorted populations. Numbers of defined clones are relative to their total number. D: Differences in propagation were observed among the sorted populations during the first 7 days in culture. Columns represent mean±SEM of three analyses. Different letters above the columns indicate statistically significant (*P*<0.05) differences. Bar = 50 µm.

Culturing MECs at low densities for clone analysis is an established method of evaluating the number and multipotency of progenitor cells [74], [75], [76], [77], [78]. In the current study, Lin^−^ cells of the four sorted bMEC populations: puStm, Basal, puPgt and Lum, were seeded at a density that allowed growth of visually distinct colonies, likely originating from a single cell and referred to as clones. On day 4 of culture, clones comprised of least 6 cells were counted and a basal vs. luminal lineage was determined according to the expression of CK14 and CK18 (Figure 5A). About 5% of the cells comprising the puStm or puPgt populations formed clones. Slightly lower potency for colony formation was measured for the Basal population. In contrast, a significantly lower number of colony-forming cells (CFCs), 60% of that determined in the puStm culture, was observed in the Lum cultures. Three clone phenotypes were identified (Figure 5B): CK14^+^CK18^−^ (basal), CK14^−^CK18^+^ (luminal) and CK14^−^CK18^−^ (other/non-epithelial, not shown). The puStm and Basal populations generated both basal and luminal clones at a ratio of 1.8∶1 and 2.5∶1, respectively (Figure 5C). In contrast, the puPgt and Lum cultures gave rise exclusively to luminal, CK18-stained clones (aside from non-epithelial clones). No significant difference was observed in the number of other/non-epithelial clones among the sorted populations. The two types of epithelial clones formed by the puStm and Basal populations might originate from bi-potent progenitors differentiating *in vitro* to either a luminal or basal phenotype. Alternatively, they may contain two types of uni-potent progenitors that originate from a less differentiated ancestor, and are committed to either the luminal or basal lineage. In contrast, the puPgt and Lum populations are limited to luminal-restricted progenitors.

A propagation-rate analysis of cells during an established period of culture may serve as a complementary tool to evaluate their hierarchical origin. Maintaining a continuous growth rate indicates top status in the hierarchy, allowing cells to exploit their “transit amplifying” stage. In contrast, cells at the bottom of the hierarchy display limited growth rate, as they are post-mitotic or near that period. bMECs of the sorted populations were seeded at a low density that enabled statistical estimation of their proliferation rate by day 7 of culture (Figure 5D). Propagation rate was calculated as the average number of cells added per hour to the culture after seeding. Interestingly, the puStm and Basal populations exhibited significantly (*P*<0.05) higher growth than the puPgt and Lum populations (*P*≤0.05), with the highest value being maintained by the puStm cells. Apparently, this population preserved the highest number of proliferating cells over this culture period. The Lum population displayed negative growth, consistent with the considerable presence of mature cells in this population.

### Repeat sorting of cultured bMEC populations

**Figure 6:**
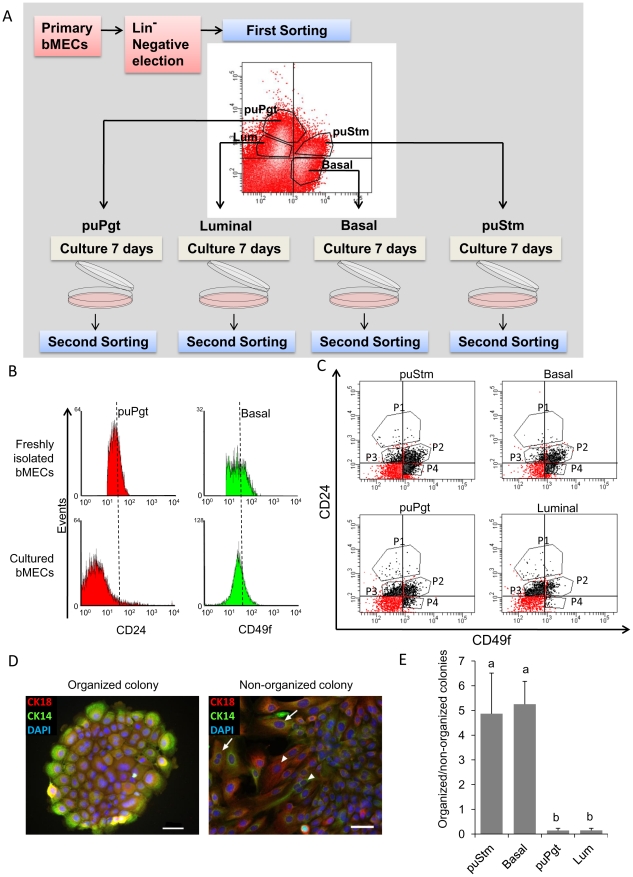
Cultured bMEC populations do not differ in their CD49f/CD24 expression, but maintain their distinct parental characteristics. A: Schematic representation of the experimental procedure. B: FACS histograms depicting the levels of CD24 and CD49f in freshly isolated bMECs compared with their cultured counterparts. C. FACS dot-plots depicting the subpopulations sorted from the cultured cells. D: Immunofluorescence staining of the lineage markers CK14 and CK18 in organized and non-organized colonies. E: Regardless their different cell-surface marker expression, organized colonies were significantly more frequent in sorted cultured cells originated from the puStm and Basal populations. Bar = 50 µm.

MaSCs differentiate *in vivo* into the full repertoire of epithelial cells that comprise the functional mammary gland [21], [22]. To test whether this ability can be recapitulated *in vitro*, cells sorted into the four populations: puStm, Basal, puPgt and Lum, were cultured separately for 7 days and sorted again according to CD24 and CD49f expression (Figure 6A). Preliminary studies established constitutive CD49f expression throughout culture, while CD24 expression was markedly reduced (Figure 6B). Thus, the second sorting was based only on distinction between CD24- and CD49f-expressing and non-expressing populations (defined by the quadrants in Figure 6C). A minority of the cells which still expressed high levels of CD24 (CD24^++^) independent of their CD49f expression were also collected, completing the four new subpopulations: CD24^++^ (P1), CD24^+^ CD49f^+^ (P2), CD24^+^CD49f^−^ (P3) and CD24^−^CD49f^+^ (P4). A population of non-epithelial CD24^−^CD49f^−^ cells was also detected but was not collected.

Subpopulations P1 through P4 were cultured separately and the resulting colonies were morphologically analyzed and stained for the lineage markers CK14 and CK18 (Figure 6D). Two types of colonies were observed: (i) organized colonies which were typically round and compact (Figure 6D, left panel). These colonies consisted of densely clustered polygonal cells expressing CK14 at the rim and CK18 at the center, thus resembling a duct-like alignment; (ii) non-organized colonies (Figure 6D, right panel) consisting of less dense, elongated cells comprising a non-descript outline and promiscuous expression of CK14 and/or CK18. Irrespective of CD24 and/or CD49f expression levels in the individual populations collected at the second sorting, most colonies (88. 9%±6.2) that developed from cells of the original puStm and Basal populations maintained an organized phenotype, while most (88.5%±6.1) of those that originated from the puPgt and Lum populations were unorganized. Taken together, these results indicated that essential properties of the puStm and Basal populations are preserved in culture, as cells of these populations assemble into an organized duct-like alignment of two distinct layers. In addition, the findings imply that CD24 and CD49f expression levels may not be useful for distinguishing cultured bMEC populations.

### Sorted bMECs form differential numbers of non-adherent colonies in culture

**Figure 7:**
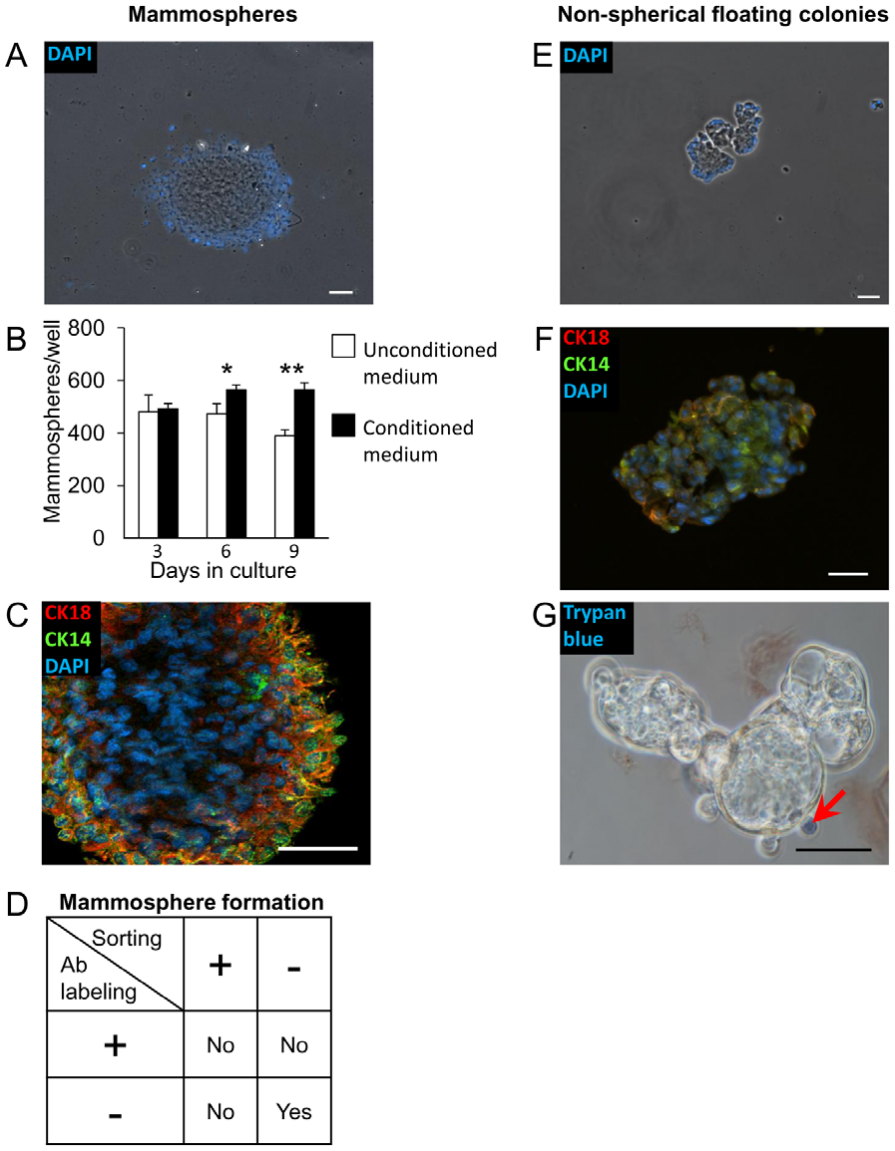
Mammospheres are formed by freshly dissociated bMECs, whereas sorting procedures induce non-spherical floating colonies (NSFCs). A: Representative mammosphere, formed by freshly dissociated Lin^−^ bMECs. Nuclei are stained with DAPI. B: Supplementation of conditioned mammary medium enhances mammosphere formation. *,**Significantly different at *P*≤0.05 and *P*≤0.01, respectively. C: The mammosphere is comprised of cells expressing CK14 and CK18. D: Antibody labeling and the sorting process prevent mammosphere formation. E: Representative NSFC formed by sorted bMECs. F: NSFC is comprised mainly of live cells. Trypan blue-stained dead cell (blue) is marked by an arrow. G: The NFSC is comprised of cells expressing CK14 and CK18. Columns represent average±SEM of three wells analyzed for each group. Bar = 50 µm.

Under non-adherent culture conditions, mammospheres develop from MaSCs that have escaped anoikis. Currently, analysis of mammosphere development is the best *in-vitro* method of evaluating stem cell frequency. Even though probably not all mammospheres are of clonal origin, the specific conditions applied enable retention of self-renewal and multipotency, as well as prolonged proliferation [23], [79]. To monitor and follow mammosphere development in the bovine mammary population, Lin^−^ bMECs were cultured under ultra-low adherence conditions and supplemented with either mammary medium or conditioned mammary medium. These cells were not stained with antibodies or sorted before culture. Visible, non-adherent mammospheres were identified on the second day of culture in both groups (Figure 7A), and a frequency of one mammosphere per 647±48 seeded Lin^−^ bMECs was estimated on day 6 for cells supplemented with mammary medium. Conditioned mammary medium marginally, but significantly (*P*<0.05) improved mammosphere frequency by day 6, and prevented the decrease in their number on day 9 of culture (Figure 7B). Epithelial origin of the mammospheres was confirmed by CK14 and CK18 staining (Figure 7C).

In contrast to the bMECs that did not undergo antibody staining or sorting, those sorted according to CD24 and CD49f expression into four populations (puStm, Basal, PuPgt and Lum) did not develop mammospheres for up to 30 days in culture, even when supplemented with conditioned mammary medium. Apparently, cell-labeling with the anti-CD24 and CD49f antibodies and the sorting process independently prevent formation of these structures (Figure 7D). Instead, non-spherical floating colonies (NSFCs) were generated (Figure 7E). The NSFCs were comprised of viable cells (Figure 7F) and expressed the epithelial markers CK14 and CK18 (Figure 7G). However, they did not preserve the rounded shape of the mammospheres.

**Figure 8:**
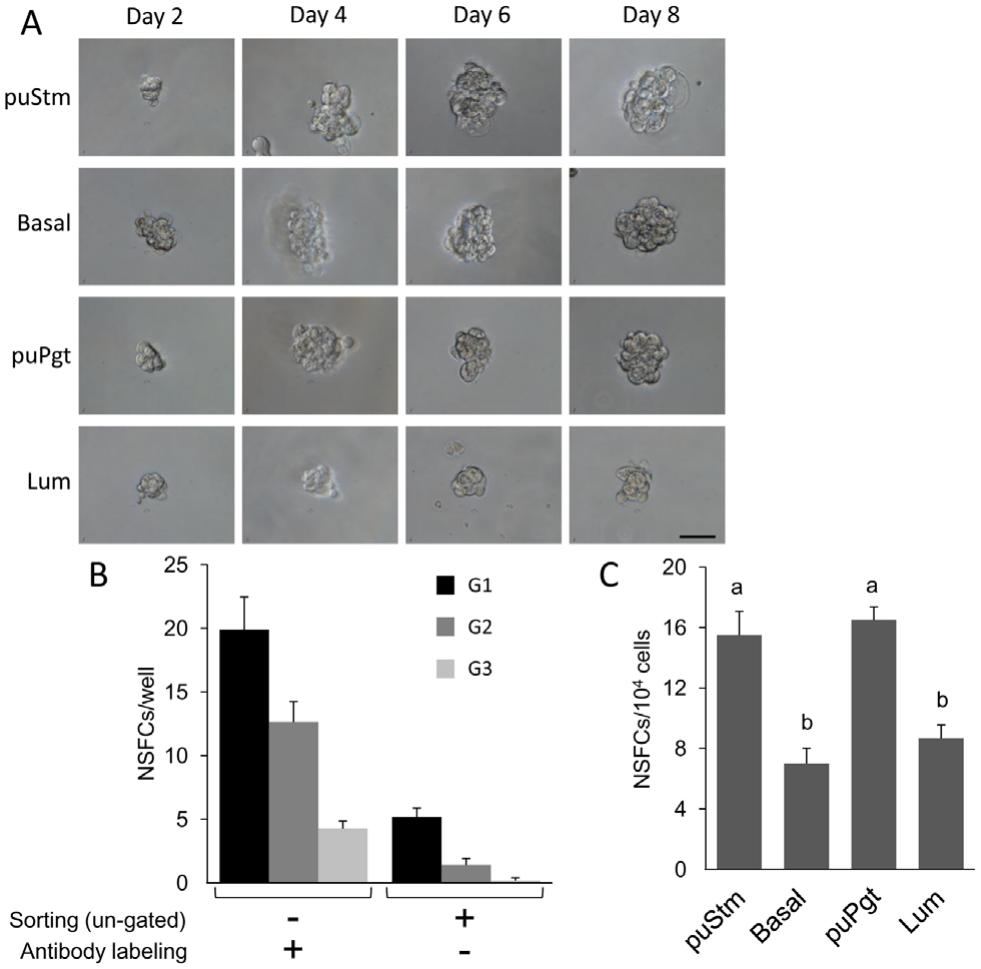
NSFC development and characteristics depend on its origin. A: Representative demonstration of limited development of a cultured NSFC from Lum cells compared to NSFCs from the other populations. B: Serial dissociation and culture of NSFCs over three generations demonstrates limited self-renewal capacity and a more severe effect of sorting, compared with antibody labeling, on their development. C: puStm and puPgt cultures generate higher numbers of NSFCs compared with Basal and Lum cultures. Columns represent mean±SEM of three analyses of least 26 floating colonies for each population. Different letters above the columns indicate statistically significant (*P*<0.05) differences. Bar = 50 µm.

Following a representative NSFC over the course of 8 days in culture revealed a limited proliferative capacity between days 2 and 6, evidenced as cell propagation and increased colony size (Figure 8A). NSFCs formed by the Lum population grew more slowly and did not reach the size of their puStm-, Basal- and puPgt-originated counterparts. This further supports the postulated enrichment of the Lum population with terminally differentiated, post-mitotic cells. Notably, expression of CK14 and CK18 was detected in NSFCs formed by all cell populations (Figure S1). Limited self-renewal capacity, which was suppressed by the sorting process, could be attributed to cells forming these non-adherent structures over three generations (Figure 8B). NSFC-forming capability varied among the Lin^−^ sorted bMEC populations (Figure 8C), being over twofold higher in the puStm and puPgt populations compared to their Basal and Lum counterparts. This difference infers higher enrichment of the puStm and puPgt populations with stem cells or their early progenitors.

### ALDH activity in bMEC populations

**Figure 9:**
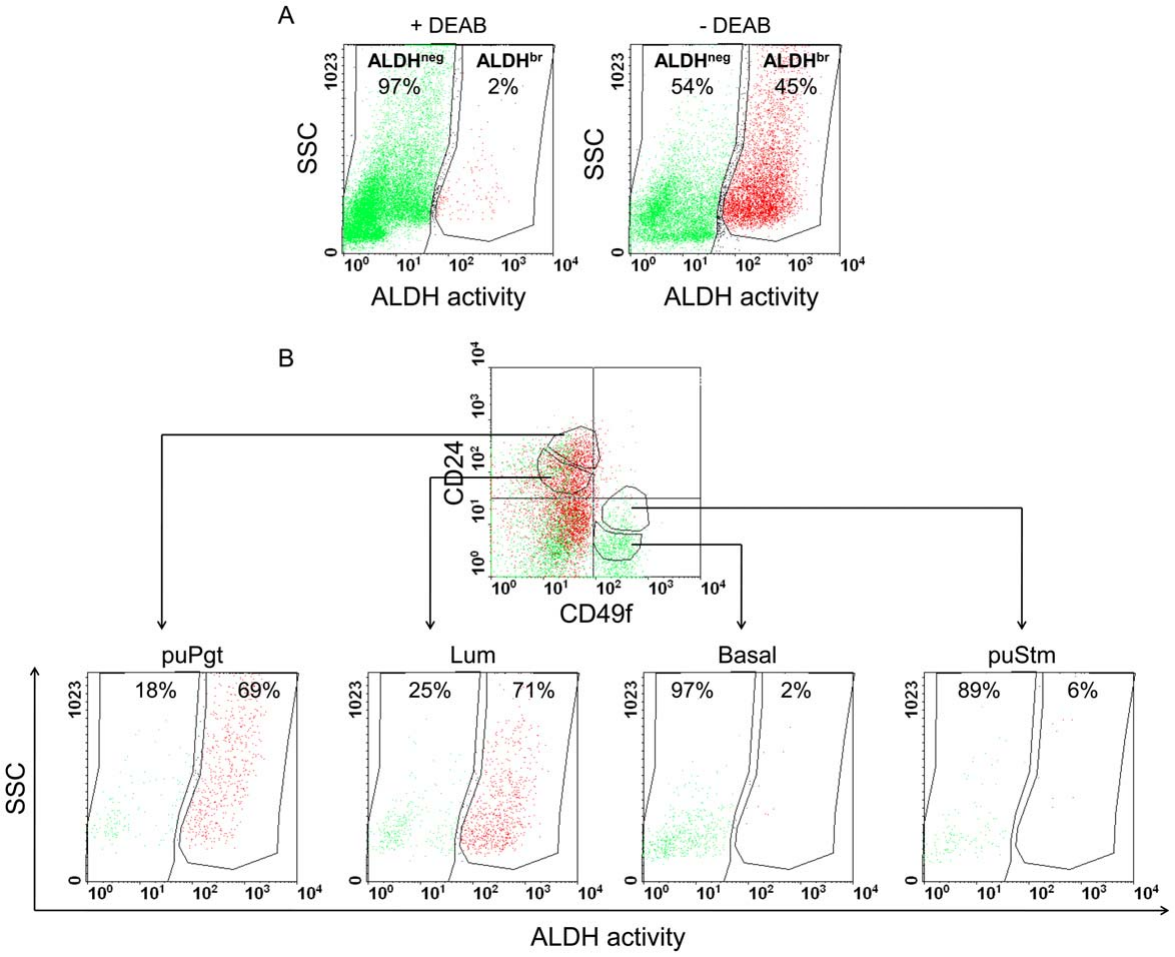
Incorporating ALDH activity into the CD24/CD49f-based analysis reveals a small ALDH^br^ population within the puStm fraction that is potentially enriched with stem cells. A: FACS analysis of Lin^−^ bMECs and gating of ALDH-positive (ALDH^br^) cells according to the effect of the ALDH inhibitor DEAB. B: Demonstration of ALDH^br^ (red) and ALDH^neg^ (green) distribution among the populations sorted according to CD49f and CD24 expression. SSC - side scatter.

The ALDEFLUOR assay enables sorting living cells according to their ALDH activity. Recently, high ALDH activity was detected in a bMEC population enriched with progenitors, while cells that were capable of regenerating epithelial structures *in vivo* were characterized by the absence of ALDH activity [43]. ALDEFLUOR-positive CD44^+^CD24^−^ Lin^−^ breast cancer cells are a small and highly tumorigenic population, putatively enriched with stem cells [69]. To study the distribution of ALDH activity among the puStm, Basal, puPgt and Lum populations, ALDEFLUOR assay was first conducted independently in Lin^−^ bMECs. A population with high ALDH activity was detected (ALDH^br^), comprising 45% of the living cells; 54% of the cells were gated as ALDH^neg^, based on a control experiment containing the ALDH inhibitor diethylaminobenzaldehyde (DEAB, Figure 9A). Clonal analysis confirmed basal and luminal characteristics of the ALDH^neg^ and ALDH^br^ populations, respectively (not shown, [43]). Merged analyses of ALDH activity and expression of cell-surface markers revealed an equal distribution of most ALDH^br^ cells among the puPgt and Lum populations, while the vast majority of the Basal and puStm populations were composed of ALDH^neg^ cells (Figure 9B). Importantly, small populations of ALDH^br^ cells, representing 2% or 6% of the Basal and puStm fractions, respectively, were also identified. These ALDH^br^ cells, especially in the puStm fraction, are potentially further enriched with cells encompassing self-renewal activity [18], [69]. Further studies to characterize these cells and confirm their stem cell properties are warranted.

## Discussion

**Figure 10:**
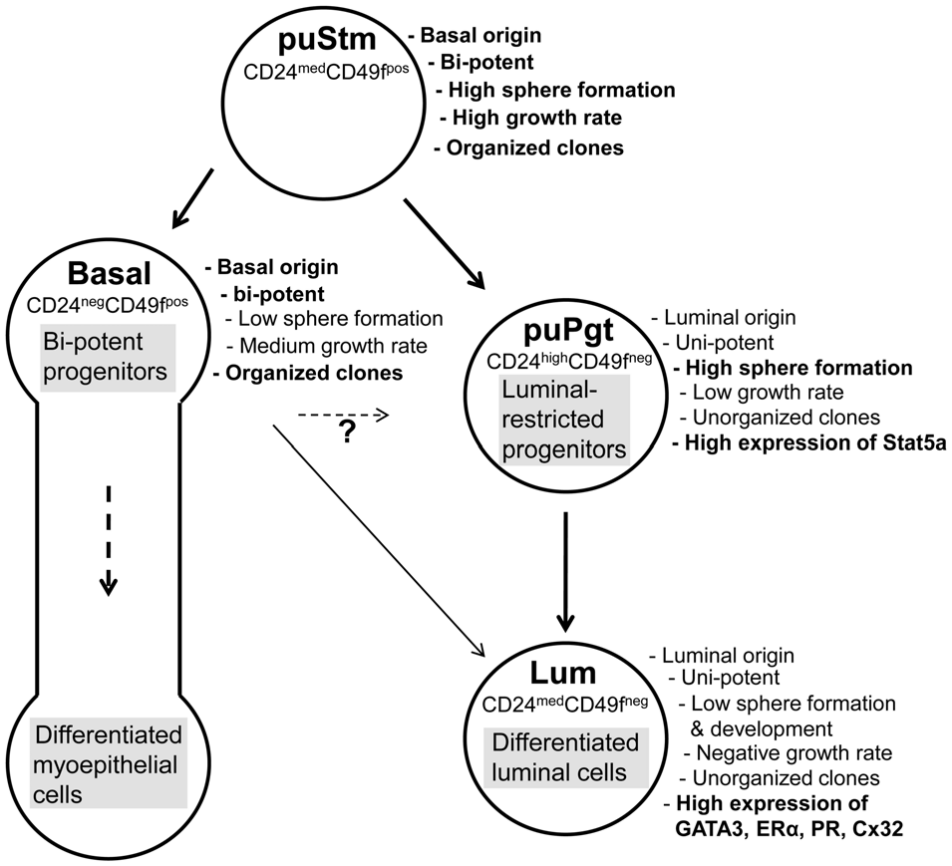
Proposed bovine mammary epithelial cell hierarchy.

The properties and hierarchy of FACS-enriched cell populations in the bovine mammary gland were studied here by measuring a spectrum of complementary capabilities. Similar to the mouse mammary gland and the human breast, the bovine mammary gland also comprises distinct populations of epithelial cells that are distinguished here for the first time according to their expression of the cell-surface markers CD24 and CD49f. *In-situ* localization of these proteins in the basal and luminal compartments of the heifer's gland, respectively, supports their use for adequate cell separation. Indeed, CD24 is also highly expressed in luminal progenitors and mature cells in the breast [59], [80], whereas low expression levels are associated with better capability of mouse MECs to regenerate new mammary ducts after transplantation (i.e. with stem cells) [59]. CD49f, on the other hand, is expressed mainly by bi-potent progenitors and basal/myoepithelial cells of the breast [53], [75] and mouse mammary gland [81], but also in some luminally restricted breast cells [53], [75]. Using these tools, bMECs were sorted, and the resulting cell-separation analysis resembled, to some extent, the distribution of the mouse mammary cells. Four populations of cells were collected (Figure 10). The puStm appears at the top of the cell hierarchy due to its basal origin, which is associated with stemness in the human breast and mouse mammary gland, and its potency to generate both basal and luminal clones. puStm cells also preserve the ability to generate organized clones with duct-like cell alignment in culture, form relatively high numbers of NSFCs and maintain the highest propagation potential *in vitro*, depicting their lowest differentiation status. Whereas in mice the ultimate evidence for the existence of mammary stem cells is clonal repopulating ability within the cleared fat pad [21], [22], such experiments are much more difficult to perform in humans [24] and bovine [82].

The Basal and puPgt populations were located downstream of the puStm population in the hierarchy. The Basal population complements the puStm population to form the basal compartment and appears higher in the hierarchy than the puPgt due to its bi-potent characteristic. However, it exhibited lower sphere formation than the puStm and retained only an intermediate propagation rate. This population might represent a mixture of bi-potent progenitors and more differentiated myoepithelial cells. Very little is known about the mechanism regulating myoepithelial cell formation and their differentiation pathway, hindering our and others' [80] attempts to dissect this lineage. Nonetheless, during the preparation of this study for publication, an elegant study in human mammary organoids was published demonstrating the involvement EGF in early expansion of myoepithelial cells via the ERK 1/2-RSK pathway, and dissecting the effect of HER1 ligands in determining the myoepithelial lineage [83]. Notably, mouse mammary organoids are much less responsive to HER1's effect than their human counterparts. This striking difference designates the bovine mammary gland as an attractive candidate for further studies of mammalian diversity in EGF-MAP kinase's regulatory role in delineating the basal/myoepithelial mammary lineage.

The puPgt population is uni-potent, as it forms only CK18-stained colonies, and does not preserve the ability to form organized structures from any tested re-sorted cell fraction. Yet its highest level of Stat5a expression particularly marks luminal progenitors [70] and its high level of sphere formation depicts its relatively high position in the cell hierarchy.

The Lum population complements the puPgt in comprising the luminal compartment and represents the lower boundary of the luminal lineage. It encompasses differentiated luminal cells expressing high levels of the luminal genes CK18, GATA3, and Cx32, as well as ERα and PR, indicating little *in-vivo* stem cell activity in mice and humans [59], [84]. These cells also exhibited low sphere formation and development, and negative propagation potential in culture. Apparently, the Lum population exhibits the characteristics of the milk-producing cells in the lactating cow that show almost no proliferation. Their gradual apoptosis during the lactation period is the main cause for decreased milk production [85].

Of note is the propagation-rate analysis that was applied here to evaluate the distance of an epithelial cell from its fully differentiated state. Once stem cells are removed from their *in-vivo* environment and seeded under adherent culture conditions, they lose their quiescent state and initiate proliferation followed by differentiation [86], [87], [88], [89]. It is this very property that warrants the development of various methods to maintain stem cells undifferentiated in culture (reviewed in [26]). The cell's *in-vitro* propagation potential is correlated with its position in the cell hierarchy: undifferentiated stem cells have to exploit more stages toward a fully differentiated state than do partly committed progenitors or post-mitotic differentiated cells. Importantly, the high propagation rate of the puStm population does not imply that it is highly proliferative *in vivo*, but rather that it is high up in the cell hierarchy.

Unlike their mouse and human counterparts, bMECs did not form mammospheres following antibody labeling or cell sorting [23], [90]. Instead, NSFCs with non-spherical morphology were formed under the non-adherent conditions. The NSFCs are composed of live, proliferating cells and preserve a degree of self-renewal, rendering them essentially comparable to mammospheres in assessing stem cell enrichment. The reason for the non-spherical organization of the labeled/sorted bMECs remains unclear. To a certain extent, antibody labeling may hinder the cell-cell interaction, particularly as one of the antibodies used here binds an integrin [22], [91]. This did not interrupt growth in 2D culture, but apparently interfered with the organization into round shapes in 3D culture. Notably, NSFCs are formed following a sorting procedure regardless of antibody labeling, raising the possibility that mechanical stress also affects membrane properties and hinders the organization of cells in a 3D culture [92]. The extent to which the labeling/sorting procedures affect successful bovine cell transplantations compared to their mouse and human counterparts has yet to be determined.

Relative expression patterns across the cell subtypes are largely conserved between the mouse mammary gland and the human breast [80]. Stem cells in both species lack ERα and PR expression and are indirectly subjected to steroid effects via the receptor activator of nuclear factor κ-B ligand (RANKL) that is secreted from neighboring ERα+/PR+ cells [32], [93] and reviewed in [84]. Thus, previous observations of some of the DNA-retaining cells in the bovine gland being ERα+ [42] are surprising. In our experiments, ERα+ and PR+ cells were scattered among the population lining the lumen. Many of them did not maintain direct contact with the lumen as shown for their human [94], but not mouse [95] counterparts. Nevertheless, none of the bovine mammary ERα+ cells acquired the basal position shown for the CD49f-expressing cells. This morphological evidence negates the basal characteristic of the mouse stem cells, but cannot rule out the presence of bovine stem cells or their immediate progenitors among the reported DNA-retaining cells.

A more distinctive characteristic of the bovine cell populations compared to mice involves localization and expression of members of the Notch pathway. This pathway regulates cell activity through a large number of factors operating in adjacent cells [96]. Notch signaling promotes self-renewal of human MaSCs and myoepithelial commitment [27], [97]. In contrast, it inhibits mouse MaSCs' self-renewal and promotes luminal commitment and proliferation [97]. In the latter, Notch activity is comprised of Notch1, Jagged1 and Hey1 expression mainly in the luminal progenitors, and basal expression of Delta1. In contrast, all four of these components were highly expressed in the basal compartments (puStm and Basal) of the bovine mammary gland. It would, therefore, be of interest to define whether and how the different expression of Notch components within the bovine and the mouse glands affects self-renewal and lineage commitment.

Identification of stem cells in adult tissues, including the mammary gland, remains difficult since much of the information regarding their actual location and fate is still elusive. Examination of the expression of stem cell markers that are indicative in other somatic tissues identified Nestin as a marker of basal/myoepithelial cells in the bovine gland, thus confirming previous findings in the human breast and mouse mammary gland [61], [62]. Lgr5, a Wnt target gene, is highly expressed in a variety of malignancies (reviewed in [64]). However, little is known about its role and expression pattern in the mammary gland. Here, immunodetection and gene-expression analyses linked Lgr5 expression to mature luminal cells of the bovine mammary gland, establishing its role as a luminal marker in this tissue. The differential expression of ALDH1 within the puStm and Basal populations infers a potentially stem cell-enriched subpopulation of basally associated cells with high ALDH activity. Indeed, combined detection of ALDH activity with surface-marker analysis identified a small candidate stem cell-enriched subpopulation, representing 0.35% of the viable sorted cells, with the expected characteristics.

In conclusion, this study demonstrates for the first time that bovine cell populations acquire the conserved cell hierarchy paradigm delineated for their mouse and human counterparts: stem cells give rise to bi-potent progenitors that differentiate along the basal/myoepithelial lineage and possibly also give rise to luminal cells. The stem cells also generate luminal-restricted progenitors that give rise to terminally differentiated cells. Importantly, some of the bovine cell properties that were involved in the definition of this hierarchy are novel or non-overlapping with mice or humans due to distinct characteristics of the bovine mammary cells. Identifying the properties of bovine stem cells and their progenitors will undoubtedly promote our understanding of the bovine mammary gland's adaptation to high and continuous milk production and its possible resistance to tumorigenesis.

## Materials and Methods

### Digestion of bovine mammary tissue into organoids

Mammary biopsies were harvested from the udders of individual 7- to 10-month-old Holstein heifers, immediately at commercial slaughter. In total, 10 heifers were analyzed. The biopsies were excised from the well-distinguished parenchymal region near the border with the mammary fat pad [41] and immediately submerged in ice-cold PBS containing 1 mg/ml streptomycin and 1000 U/ml penicillin (Biological Industries, Beit Haemek, Israel) for 20 min. The supplementation of a 10-fold higher antibiotic concentration relative to culture conditions was essential to prevent bacterial contamination. Study protocols were in compliance with the regulations of the Israeli Ministry of Health and the Volcani Center's institutional policies (approval no. IL- 202/09). Preparation of organoids from the bovine mammary tissue followed the protocol established by Proia and Kuperwasser [19] for their organoid preparation from human breast tissue with some modifications. Briefly, the fatty tissue was removed and 3- to 5-g pieces of the remaining parenchyma were minced with fine scissors into 3- to 5-mm^3^ pieces. These pieces were digested at 37°C for 3 h in slowly shaken 50-ml conical tubes containing 10 ml DMEM-F12 medium (Biological Industries) supplemented with 5% fetal bovine serum (FBS, Biological Industries), type II collagenase (300 U/ml, Worthington, Lakewood, NJ), hyaluronidase (100 U/ml, Sigma, St. Louis, MO), insulin (5 µg/ml, Sigma) and hydrocortisone (1 µg/ml, Sigma). The 3-h digestion period was calibrated to obtain an optimal ratio of viable to total number of dissociated cells. The resulting organoids were washed in PBS containing 5% FBS and aliquots were stored at −80°C in FBS supplemented with 10% DMSO.

### Dissociation of bovine mammary organoids into single-cell suspension

Organoids were washed in HBSS (Biological Industries) containing 5% FBS (HF) and treated for 3 min first with trypsin-EDTA solution (Biological Industries) and then with dispase enzymatic solution (50 caseinolytic units/ml, BD Biosciences, Bedford, MA) that contained DNAse-I (0.125 mg/ml, Worthington). The dissociated cells were washed, resuspended in HF and separated from tissue debris and cell aggregates by filtration through a metal mesh followed by a cell strainer (BD Falcon, Bedford, MA) with pores of 70 µm and 40 µm diameter, respectively.

### Flow cytometry

Lin^−^ cell suspension was prepared using the EasySep mouse mammary enrichment kit (StemCell Technologies, Vancouver, Canada) according to the manufacturer's protocol. Antibodies to the mouse hematopoietic cell-surface antigens CD45, CD31 and TER119 allowed elimination of the bovine hematopoietic cells due to their relatively conserved antigen sequences (78% and 77% homology between mouse and bovine for CD45 and CD31, respectively). Epithelial cells were resuspended in HF (10^7^ cells/ml) and incubated for 120 min on ice with PE-conjugated anti-CD24 and FITC-conjugated anti-CD49f antibodies (Table 1). PI (Sigma) staining was performed to mark dead cells, and cell clumps were excluded by filtration through the metal mesh. Cell sorting and cell analyses were performed in a FACSAria II cell sorter and LSR II flow cytometer (BD Biosciences), respectively, at the Department of Biological Services of the Weizmann Institute of Science (Rehovot, Israel). Resulting data were visualized and analyzed by FACSDiva (BD Biosciences) and WinMDI 2.9 (Scripps Research Institute, La Jolla, CA) software. Determination of ALDH activity by cell sorting was performed after exclusion of dead cells and debris using the ALDEFLUOR kit (StemCell Technologies) according to the manufacturer's protocol. The kit allows visualization of ALDH activity with the green fluorescence channel (520–540 nm). To combine the sorting according to CD24 and CD49f expression with ALDH activity, anti-CD49f antibodies conjugated to PE-Cy5 fluorophore were used (BD Biosciences) instead of anti CD49f-FITC, at the same dilution, thus enabling detection of ALDH and the surface markers through different channels.

**Table 1:** List of antibodies used in the study.

Antigen	Primary antibody	Manufacturer	Dilution	Secondary antibody	Manufacturer	Dilution
ALDH1	Mouse monoclonal, clone 44	BD Transduction Laboratories, Bedford, MA	1∶50	Labeled polymer-HRP anti-mouse	Dako, Glostrup, Denmark	1∶1
CD24	PE-conjugated, rat monoclonal, clone M1/69	StemCell Technologies, Vancouver, Canada	1∶30	Not applied	Not applied	Notapplied
CD49f	FITC-conjugated, rat monoclonal, clone GoH3	StemCell Technologies	1∶20	Not applied	Not applied	Not applied
CK14	Mouse monoclonal, clone LL002	AbD Serotec, Oxford, UK	1∶50	Cy2-conjugated goat anti-mouse IgG	Jackson ImmunoResearch, West Grove, PA	1∶200
CK18	Chicken polyclonal, ab14047	Abcam, Cambridge, UK	1∶130	Alexa Fluor 555-conjugated goat anti-chicken IgG	Molecular Probes, Eugene, OR	1∶1000
CK6	Mouse monoclonal, clone LHK6	Santa Cruz Biotechnology, Santa Cruz, CA	1∶50	Cy3-conjugated donkey anti-mouse IgG	Jackson ImmunoResearch	1∶200
Delta1	Mouse monoclonal, ID 35663	LifeSpan BioSciences, Seattle, WA	1∶50	Cy3-conjugated donkey anti-mouse IgG	Jackson ImmunoResearch	1∶200
ERα	Rabbit polyclonal, H-184	Santa Cruz Biotechnology	1∶50	Alexa Fluor 488-conjugated goat anti-rabbit IgG	Molecular Probes	1∶500
GATA3	Mouse monoclonal, clone HG3-31	Santa Cruz Biotechnology	1∶50	Cy3-conjugated donkey anti-mouse IgG	Jackson ImmunoResearch	1∶200
Lgr5	Rabbit polyclonal, LS-C98616	LifeSpan BioSciences	1∶10	Alexa Fluor 488-conjugated goat anti-rabbit IgG	Molecular Probes	1∶500
Nestin	Mouse monoclonal, clone Rat-401	Cell Signaling Technology, Beverly, MA	1∶30	Cy3-conjugated donkey anti-mouse IgG	Jackson ImmunoResearch	1∶200
Notch1	Rabbit polyclonal, H-131	Santa Cruz Biotechnology	1∶50	Alexa Fluor 488-conjugated goat anti-rabbit IgG	Molecular Probes	1∶500
p63	Rabbit polyclonal, H-137	Santa Cruz Biotechnology	1∶50	Cy3-conjugated donkey anti-rabbit IgG	Jackson ImmunoResearch	1∶200
PR	Mouse monoclonal, clone Alpha PR6	Abcam	1∶50	Cy3-conjugated donkey anti-mouse IgG	Jackson ImmunoResearch	1∶200
αSMA	Mouse monoclonal, IgG2a kappa, clone 1A4	Dako	1∶50	Cy3-conjugated donkey anti-mouse IgG	Jackson ImmunoResearch	1∶200

### Histological analysis and immunostaining

For immunostaining of cells in culture, 4% paraformaldehyde-fixed cells were washed with PBS and treated with 0.5% Triton X-100 (BDH, Poole, England) for 5 min. Following overnight incubation in blocking solution (2% goat serum and 1% BSA in PBS) at 4°C, the fixed cultures were reacted with primary antibodies for 1 h at room temperature and then overnight at 4°C. Incubation with secondary antibodies proceeded for 1 h at room temperature, and nuclei were stained with DAPI (Qbiogen, Irvine, CA). The antibodies and their dilutions are listed in Table 1.

Tissue immunostaining was performed on either paraffin-embedded or frozen tissue sections. For paraffin-embedded sections, biopsies were fixed in Bouin's solution, dehydrated in a graded ethanol series (50% to 100%), cleared in xylene and embedded in paraffin. For frozen tissue sections, biopsies were fixed in 4% paraformaldehyde solution containing 1% sucrose for 2 h at room temperature and then incubated in a series of paraformaldehyde solutions containing 5%, 10% and 20% sucrose, each for 30 min. Finally, the biopsies were incubated overnight at 4°C in paraformaldehyde solution containing 30% sucrose. Tissues were submerged in O.C.T compound (Sakura Finetek, Alphen aan den Rijn, The Netherlands), frozen in liquid nitrogen and stored at −80°C.

Immunostaining was performed on 5-µm paraffin-embedded or frozen sections. Antigen retrieval was performed on all sections by boiling in 0.01 M citrate buffer for 10 min. The reactions with primary and secondary fluorescence-labeled antibodies (Table 1) followed the protocol described for cultured cells. For immunohistochemistry, paraffin-embedded sections were treated with 3% hydrogen peroxide for 30 min and boiled for 10 min in 0.01 M citrate. Sections were incubated with the primary antibody (Table 1), followed by incubation with EnVision-labeled HRP polymer (DakoCytomation, Glostrup, Denmark) for 1 h at room temperature. Signal was generated with 3,3′-diaminobenzidine (DAB) substrate kit (Vector Laboratories, Burlingame, CA) according to the manufacturer's protocol and counterstaining was performed with hematoxylin (Sigma).

### RNA extraction and real-time PCR analysis

RNA was extracted from sorted cell populations using RNeasy Plus Micro kit (Qiagen, Hilden, Germany) according to the manufacturer's protocol. Quantitative real-time PCR analyses were performed in a StepOnePlus instrument (Applied Biosystems, Foster City, CA) in a 20-µl reaction volume containing 5 µl cDNA, 10 µl SYBR Green fast PCR Master Mix (Applied Biosystems) and 10 µM primers (Table S1). The thermal-cycling conditions consisted of 20 s at 95°C followed by 40 cycles of 3 s at 95°C and 30 s at 60°C. The primers were designed to yield a single product without primer dimerization, and across exon-exon junctions. The amplification curves for the selected genes were parallel. Hprt1 and 16 S were used together as control genes and expression levels were calculated using StepOne v2.1 or DataAssist v2.0 software (Applied Biosystems), relative to the total, ungated population. Results represent the average±SEM of four biological repeats, and statistical significance was calculated by paired t-tests comparing each population to its three counterparts.

### Clonal assay

Cells were sorted into populations according to the expression levels of CD24 and CD49f and seeded in 24-well culture plates (Corning, Lowell, MA) at a density of 5000 cells/well (2631 cells/cm^2^) according to events counted by the FACS sorter. Cells were cultured for 3 days in DMEM-F12 medium containing 5% FBS, hydrocortisone (0.5 µg/ml, Sigma), insulin (5 µg/ml, Sigma), gentamicin (50 µg/ml, Biological Industries), streptomycin and penicillin (100 µg/ml and 100 U/ml, respectively), hEGF (10 ng/ml, Merck, Darmstadt, Germany), hFGF2 (10 ng/ml, Merck), heparin (4 µg/ml, Merck), cholera toxin (10 ng/ml, Sigma) and B27 (4 µl stock/ml, Invitrogen, Carlsbad, CA). This medium was termed “mammary medium”. Developing clones were fixed in 4% paraformaldehyde supplemented with 0.03 M sucrose, permeabilized with 0.5% Triton X-100 and stained with antibodies to p63, CK14 or CK18 (Table 1) as described in the Histological analysis and immunostaining section. Clones consisting of at least six individual adjacent cells were counted using an inverted fluorescence microscope (Eclipse Ti, Nikon Instruments, Melville, NY) and characterized as luminal, basal or other according to the expression of the lineage markers.

### Analysis of propagation rate

Cells from each of the sorted populations were seeded in six-well cell-culture plates (Corning) at a density of 10,000 cells/well (1052 cells/cm^2^) and cultured in mammary medium for 7 days. The difference in cell number (Δ) was calculated as (N_2_−N_1_)/(t_2_−t_1_), where N_1_ and N_2_ represent the number of cells counted on day of seeding (t_1_) and after 7 days (t_2_), respectively.

### Repeat sorting of cultured cell populations

Sorted bMEC populations were cultured in six-well cell-culture plates (Corning) at a density of 25,000 cells/well (2631 cells/cm^2^) and supplemented with mammary medium. Cells were harvested on day 7, washed in HF and stained with PE-conjugated anti-CD24 and FITC-conjugated anti-CD49f antibodies, as described for the flow cytometry. Gated populations were sorted, seeded in 24-well plates (Corning) at a density of 5000 cells/well (2631 cells/cm^2^) and cultured in mammary medium. After 3 days, the colonies were fixed and stained as described for histological analysis and immunostaining.

### Mammosphere assay

Sorted bMEC populations were individually seeded in 96- or 6-well ultra-low-attachment plates (Corning) at a density of 100 cells/µl (10,000 cells/well) and supplemented with mammary medium or conditioned mammary medium (mammary medium harvested after incubation with bMECs for 5 days and mixed 1∶1 with fresh mammary medium). Developing mammospheres were dissociated into a single-cell suspension using trypsin-EDTA and cells were collected by centrifugation at 663 *g* for 5 min. For fixation, mammospheres and NSFCs were collected by centrifugation at 663 *g* for 5 min and resuspended in PBS. The suspension was placed on warm glass slides on a hot plate adjusted to 37°C until the liquid evaporated and the mammospheres/NSFCs were visible. Trypsin was briefly added to the warm slides to loosen cell-cell contact and allow subsequent antibody access [25]. Slides were then washed in PBS and the mammospheres/NSFCs were fixed in an ice-cold, 1∶1 methanol-acetone solution for 10 min. For immunostaining, slides were washed in PBS, incubated in blocking solution containing 0.5% Triton X-100 for 1 h at room temperature and subjected to CK14 or CK18 antibodies as described for histological analysis and immunostaining. Mammospheres or NSFCs were visualized using an inverted fluorescence microscope (Eclipse Ti) or Olympus IX 81inverted laser scanning confocal microscope (FLUOVIEW 500, Tokyo, Japan).

## Supporting Information

Figure S1 **Immunofluorescence staining of single representative NSFCs formed by each sorted population**. Bar = 50 µm.
(TIF)

Table S1 **List of primers used to amplify coding regions of the listed genes.**
(DOCX)

## References

[1] Molyneux G, Regan J, Smalley MJ (2007). Mammary stem cells and breast cancer.. Cell Mol Life Sci.

[2] Deome KB, Faulkin LJ, Bern HA, Blair PB (1959). Development of mammary tumors from hyperplastic alveolar nodules transplanted into gland-free mammary fat pads of female C3H mice.. Cancer Res.

[3] Daniel CW, De Ome KB, Young JT, Blair PB, Faulkin LJ (1968). The in vivo life span of normal and preneoplastic mouse mammary glands: a serial transplantation study.. Proc Natl Acad Sci U S A.

[4] Hoshino K, Gardner WU (1967). Transplantability and life span of mammary gland during serial transplantation in mice.. Nature.

[5] Smith GH, Medina D (1988). A morphologically distinct candidate for an epithelial stem cell in mouse mammary gland.. J Cell Sci.

[6] Woodward WA, Chen MS, Behbod F, Rosen JM (2005). On mammary stem cells.. J Cell Sci.

[7] Ferguson DJ (1988). An ultrastructural study of mitosis and cytokinesis in normal ‘resting’ human breast.. Cell Tissue Res.

[8] Chepko G, Smith GH (1997). Three division-competent, structurally-distinct cell populations contribute to murine mammary epithelial renewal.. Tissue Cell.

[9] Welm BE, Tepera SB, Venezia T, Graubert TA, Rosen JM (2002). Sca-1(pos) cells in the mouse mammary gland represent an enriched progenitor cell population.. Dev Biol.

[10] Smalley MJ, Titley I, Ashworth A (2005). An improved definition of mouse mammary epithelial side population cells.. Cytotherapy.

[11] Alvi AJ, Clayton H, Joshi C, Enver T, Ashworth A (2003). Functional and molecular characterisation of mammary side population cells.. Breast Cancer Res.

[12] Zeps N, Dawkins HJ, Papadimitriou JM, Redmond SL, Walters MI (1996). Detection of a population of long-lived cells in mammary epithelium of the mouse.. Cell Tissue Res.

[13] Zeps N, Bentel JM, Papadimitriou JM, D'Antuono MF, Dawkins HJ (1998). Estrogen receptor-negative epithelial cells in mouse mammary gland development and growth.. Differentiation.

[14] Smith GH (2005). Label-retaining epithelial cells in mouse mammary gland divide asymmetrically and retain their template DNA strands.. Development.

[15] Clarke RB, Spence K, Anderson E, Howell A, Okano H (2005). A putative human breast stem cell population is enriched for steroid receptor-positive cells.. Dev Biol.

[16] Clarke RB, Anderson E, Howell A, Potten CS (2003). Regulation of human breast epithelial stem cells.. Cell Prolif.

[17] Booth BW, Smith GH (2006). Estrogen receptor-alpha and progesterone receptor are expressed in label-retaining mammary epithelial cells that divide asymmetrically and retain their template DNA strands.. Breast Cancer Res.

[18] Stingl J (2009). Detection and analysis of mammary gland stem cells.. J Pathol.

[19] Proia DA, Kuperwasser C (2006). Reconstruction of human mammary tissues in a mouse model.. Nat Protoc.

[20] Kuperwasser C, Chavarria T, Wu M, Magrane G, Gray JW (2004). Reconstruction of functionally normal and malignant human breast tissues in mice.. Proc Natl Acad Sci U S A.

[21] Shackleton M, Vaillant F, Simpson KJ, Stingl J, Smyth GK (2006). Generation of a functional mammary gland from a single stem cell.. Nature.

[22] Stingl J, Eirew P, Ricketson I, Shackleton M, Vaillant F (2006). Purification and unique properties of mammary epithelial stem cells.. Nature.

[23] Dontu G, Abdallah WM, Foley JM, Jackson KW, Clarke MF (2003). In vitro propagation and transcriptional profiling of human mammary stem/progenitor cells.. Genes Dev.

[24] Villadsen R, Fridriksdottir AJ, Ronnov-Jessen L, Gudjonsson T, Rank F (2007). Evidence for a stem cell hierarchy in the adult human breast.. J Cell Biol.

[25] Dey D, Saxena M, Paranjape AN, Krishnan V, Giraddi R (2009). Phenotypic and functional characterization of human mammary stem/progenitor cells in long term culture.. PLoS One.

[26] Dontu G, Wicha MS (2005). Survival of mammary stem cells in suspension culture: implications for stem cell biology and neoplasia.. J Mammary Gland Biol Neoplasia.

[27] Dontu G, Jackson KW, McNicholas E, Kawamura MJ, Abdallah WM (2004). Role of Notch signaling in cell-fate determination of human mammary stem/progenitor cells.. Breast Cancer Res.

[28] Grudzien P, Lo S, Albain KS, Robinson P, Rajan P (2010). Inhibition of Notch signaling reduces the stem-like population of breast cancer cells and prevents mammosphere formation.. Anticancer Res.

[29] Yan XL, Fu CJ, Chen L, Qin JH, Zeng Q (2011). Mesenchymal stem cells from primary breast cancer tissue promote cancer proliferation and enhance mammosphere formation partially via EGF/EGFR/Akt pathway.. Breast Cancer Res Treat.

[30] Paranjape AN, Mandal T, Mukherjee G, Kumar MV, Sengupta K (2011). Introduction of SV40ER and hTERT into mammospheres generates breast cancer cells with stem cell properties.. Oncogene.

[31] Tiede BJ, Owens LA, Li F, DeCoste C, Kang Y (2009). A novel mouse model for non-invasive single marker tracking of mammary stem cells in vivo reveals stem cell dynamics throughout pregnancy.. PLoS One.

[32] Asselin-Labat ML, Vaillant F, Sheridan JM, Pal B, Wu D (2010). Control of mammary stem cell function by steroid hormone signalling.. Nature.

[33] Matulka LA, Triplett AA, Wagner KU (2007). Parity-induced mammary epithelial cells are multipotent and express cell surface markers associated with stem cells.. Dev Biol.

[34] Wagner KU, Boulanger CA, Henry MD, Sgagias M, Hennighausen L (2002). An adjunct mammary epithelial cell population in parous females: its role in functional adaptation and tissue renewal.. Development.

[35] Britt KL, Kendrick H, Regan JL, Molyneux G, Magnay FA (2009). Pregnancy in the mature adult mouse does not alter the proportion of mammary epithelial stem/progenitor cells.. Breast Cancer Res.

[36] Siwko SK, Dong J, Lewis MT, Liu H, Hilsenbeck SG (2008). Evidence that an early pregnancy causes a persistent decrease in the number of functional mammary epithelial stem cells–implications for pregnancy-induced protection against breast cancer.. Stem Cells.

[37] Bai L, Rohrschneider LR (2010). s-SHIP promoter expression marks activated stem cells in developing mouse mammary tissue.. Genes Dev.

[38] Tiede B, Kang Y (2011). From milk to malignancy: the role of mammary stem cells in development, pregnancy and breast cancer.. Cell Res.

[39] Ellis S, Capuco AV (2002). Cell proliferation in bovine mammary epithelium: identification of the primary proliferative cell population.. Tissue Cell.

[40] Holland MS, Tai MH, Trosko JE, Griffin LD, Stasko JA (2003). Isolation and differentiation of bovine mammary gland progenitor cell populations.. Am J Vet Res.

[41] Capuco AV (2007). Identification of putative bovine mammary epithelial stem cells by their retention of labeled DNA strands.. Exp Biol Med (Maywood).

[42] Capuco AV, Evock-Clover CM, Minuti A, Wood DL (2009). In vivo expansion of the mammary stem/progenitor cell population by xanthosine infusion.. Exp Biol Med (Maywood).

[43] Martignani E, Eirew P, Accornero P, Eaves CJ, Baratta M (2010). Human milk protein production in xenografts of genetically engineered bovine mammary epithelial stem cells.. PLoS One.

[44] Sheffield LG (1988). Organization and growth of mammary epithelia in the mammary gland fat pad.. J Dairy Sci.

[45] Hovey RC, McFadden TB, Akers RM (1999). Regulation of mammary gland growth and morphogenesis by the mammary fat pad: a species comparison.. J Mammary Gland Biol Neoplasia.

[46] Neville MC, Medina D, Monks J, Hovey RC (1998). The mammary fat pad.. J Mammary Gland Biol Neoplasia.

[47] Capuco AV, Wood DL, Baldwin R, McLeod K, Paape MJ (2001). Mammary cell number, proliferation, and apoptosis during a bovine lactation: relation to milk production and effect of bST.. J Dairy Sci.

[48] Loor JJ, Cohick WS (2009). ASAS centennial paper: Lactation biology for the twenty-first century.. J Anim Sci.

[49] Cardiff RD, Wellings SR (1999). The comparative pathology of human and mouse mammary glands.. J Mammary Gland Biol Neoplasia.

[50] Cardiff RD (1998). Are the TDLU of the human the same as the LA of mice?. J Mammary Gland Biol Neoplasia.

[51] Hellmen E, Isaksson A (1997). Immunohistochemical investigation into the distribution pattern of myoepithelial cells in the bovine mammary gland.. J Dairy Res.

[52] Alkafafy M, Rashed R, Helal A (2011). Immunohistochemical studies on the bovine lactating mammary gland (Bos taurus).. Acta Histochem..

[53] Koukoulis GK, Virtanen I, Korhonen M, Laitinen L, Quaranta V (1991). Immunohistochemical localization of integrins in the normal, hyperplastic, and neoplastic breast. Correlations with their functions as receptors and cell adhesion molecules.. Am J Pathol.

[54] Jones C, Mackay A, Grigoriadis A, Cossu A, Reis-Filho JS (2004). Expression profiling of purified normal human luminal and myoepithelial breast cells: identification of novel prognostic markers for breast cancer.. Cancer Res.

[55] Cremers N, Deugnier MA, Sleeman JP (2010). Loss of CD24 expression promotes ductal branching in the murine mammary gland.. Cell Mol Life Sci.

[56] Buono KD, Robinson GW, Martin C, Shi S, Stanley P (2006). The canonical Notch/RBP-J signaling pathway controls the balance of cell lineages in mammary epithelium during pregnancy.. Dev Biol.

[57] Smith GH, Mehrel T, Roop DR (1990). Differential keratin gene expression in developing, differentiating, preneoplastic, and neoplastic mouse mammary epithelium.. Cell Growth Differ.

[58] Sleeman KE, Kendrick H, Robertson D, Isacke CM, Ashworth A (2007). Dissociation of estrogen receptor expression and in vivo stem cell activity in the mammary gland.. J Cell Biol.

[59] Sleeman KE, Kendrick H, Ashworth A, Isacke CM, Smalley MJ (2006). CD24 staining of mouse mammary gland cells defines luminal epithelial, myoepithelial/basal and non-epithelial cells.. Breast Cancer Res.

[60] Asselin-Labat ML, Sutherland KD, Barker H, Thomas R, Shackleton M (2007). Gata-3 is an essential regulator of mammary-gland morphogenesis and luminal-cell differentiation.. Nat Cell Biol.

[61] Li H, Cherukuri P, Li N, Cowling V, Spinella M (2007). Nestin is expressed in the basal/myoepithelial layer of the mammary gland and is a selective marker of basal epithelial breast tumors.. Cancer Res.

[62] Kolar Z, Ehrmann J, Turashvili G, Bouchal J, Mokry J (2007). A novel myoepithelial/progenitor cell marker in the breast?. Virchows Arch.

[63] Cregan MD, Fan Y, Appelbee A, Brown ML, Klopcic B (2007). Identification of nestin-positive putative mammary stem cells in human breastmilk.. Cell Tissue Res.

[64] Barker N, Clevers H (2010). Leucine-rich repeat-containing G-protein-coupled receptors as markers of adult stem cells.. Gastroenterology.

[65] Barker N, van Es JH, Kuipers J, Kujala P, van den Born M (2007). Identification of stem cells in small intestine and colon by marker gene Lgr5.. Nature.

[66] Jaks V, Barker N, Kasper M, van Es JH, Snippert HJ (2008). Lgr5 marks cycling, yet long-lived, hair follicle stem cells.. Nat Genet.

[67] Hsu SY, Liang SG, Hsueh AJ (1998). Characterization of two LGR genes homologous to gonadotropin and thyrotropin receptors with extracellular leucine-rich repeats and a G protein-coupled, seven-transmembrane region.. Mol Endocrinol.

[68] Armstrong L, Stojkovic M, Dimmick I, Ahmad S, Stojkovic P (2004). Phenotypic characterization of murine primitive hematopoietic progenitor cells isolated on basis of aldehyde dehydrogenase activity.. Stem Cells.

[69] Ginestier C, Hur MH, Charafe-Jauffret E, Monville F, Dutcher J (2007). ALDH1 is a marker of normal and malignant human mammary stem cells and a predictor of poor clinical outcome.. Cell Stem Cell.

[70] Yamaji D, Na R, Feuermann Y, Pechhold S, Chen W (2009). Development of mammary luminal progenitor cells is controlled by the transcription factor STAT5A.. Genes Dev.

[71] Miyoshi K, Shillingford JM, Smith GH, Grimm SL, Wagner KU (2001). Signal transducer and activator of transcription (Stat) 5 controls the proliferation and differentiation of mammary alveolar epithelium.. J Cell Biol.

[72] Barash I (2006). Stat5 in the mammary gland: controlling normal development and cancer.. J Cell Physiol.

[73] Reichenstein M, Rauner G, Barash I (2011). Conditional repression of STAT5 expression during lactation reveals its exclusive roles in mammary gland morphology, milk-protein gene expression, and neonate growth.. Mol Reprod Dev.

[74] O'Hare MJ, Ormerod MG, Monaghan P, Lane EB, Gusterson BA (1991). Characterization in vitro of luminal and myoepithelial cells isolated from the human mammary gland by cell sorting.. Differentiation.

[75] Stingl J, Eaves CJ, Zandieh I, Emerman JT (2001). Characterization of bipotent mammary epithelial progenitor cells in normal adult human breast tissue.. Breast Cancer Res Treat.

[76] Dundas SR, Ormerod MG, Gusterson BA, O'Hare MJ (1991). Characterization of luminal and basal cells flow-sorted from the adult rat mammary parenchyma.. J Cell Sci.

[77] Smalley MJ, Titley J, O'Hare MJ (1998). Clonal characterization of mouse mammary luminal epithelial and myoepithelial cells separated by fluorescence-activated cell sorting.. In Vitro Cell Dev Biol Anim.

[78] Stingl J, Eaves CJ, Kuusk U, Emerman JT (1998). Phenotypic and functional characterization in vitro of a multipotent epithelial cell present in the normal adult human breast.. Differentiation.

[79] Soule HD, McGrath CM (1986). A simplified method for passage and long-term growth of human mammary epithelial cells.. In Vitro Cell Dev Biol.

[80] Lim E, Wu D, Pal B, Bouras T, Asselin-Labat ML (2010). Transcriptome analyses of mouse and human mammary cell subpopulations reveal multiple conserved genes and pathways.. Breast Cancer Res.

[81] Stingl J, Raouf A, Eirew P, Eaves CJ (2006). Deciphering the mammary epithelial cell hierarchy.. Cell Cycle.

[82] Sheffield LG, Welsch CW (1986). Transplantation of bovine mammary tissue to athymic nude mice: growth subcutaneously and in mammary gland-free fat pads.. J Dairy Sci.

[83] Pasic L, Eisinger-Mathason TS, Velayudhan BT, Moskaluk CA, Brenin DR (2011). Sustained activation of the HER1-ERK1/2-RSK signaling pathway controls myoepithelial cell fate in human mammary tissue.. Genes Dev.

[84] Stingl J (2011). Estrogen and progesterone in normal mammary gland development and in cancer.. Horm Cancer.

[85] Capuco AV, Ellis SE, Hale SA, Long E, Erdman RA (2003). Lactation persistency: insights from mammary cell proliferation studies.. J Anim Sci.

[86] Muschler J, Lochter A, Roskelley CD, Yurchenco P, Bissell MJ (1999). Division of labor among the alpha6beta4 integrin, beta1 integrins, and an E3 laminin receptor to signal morphogenesis and beta-casein expression in mammary epithelial cells.. Mol Biol Cell.

[87] Reynolds BA, Weiss S (1996). Clonal and population analyses demonstrate that an EGF-responsive mammalian embryonic CNS precursor is a stem cell.. Dev Biol.

[88] Romanov SR, Kozakiewicz BK, Holst CR, Stampfer MR, Haupt LM (2001). Normal human mammary epithelial cells spontaneously escape senescence and acquire genomic changes.. Nature.

[89] Simian M, Hirai Y, Navre M, Werb Z, Lochter A (2001). The interplay of matrix metalloproteinases, morphogens and growth factors is necessary for branching of mammary epithelial cells.. Development.

[90] Liao MJ, Zhang CC, Zhou B, Zimonjic DB, Mani SA (2007). Enrichment of a population of mammary gland cells that form mammospheres and have in vivo repopulating activity.. Cancer Res.

[91] Taddei I, Deugnier MA, Faraldo MM, Petit V, Bouvard D (2008). Beta1 integrin deletion from the basal compartment of the mammary epithelium affects stem cells.. Nat Cell Biol.

[92] Jean C, Gravelle P, Fournie JJ, Laurent G (2011). Influence of stress on extracellular matrix and integrin biology.. Oncogene.

[93] Joshi PA, Jackson HW, Beristain AG, Di Grappa MA, Mote PA Progesterone induces adult mammary stem cell expansion.. Nature.

[94] Li S, Han B, Liu G, Li S, Ouellet J (2010). Immunocytochemical localization of sex steroid hormone receptors in normal human mammary gland.. J Histochem Cytochem.

[95] Shyamala G, Chou YC, Louie SG, Guzman RC, Smith GH (2002). Cellular expression of estrogen and progesterone receptors in mammary glands: regulation by hormones, development and aging.. J Steroid Biochem Mol Biol.

[96] Artavanis-Tsakonas S, Rand MD, Lake RJ (1999). Notch signaling: cell fate control and signal integration in development.. Science.

[97] Bouras T, Pal B, Vaillant F, Harburg G, Asselin-Labat ML (2008). Notch signaling regulates mammary stem cell function and luminal cell-fate commitment.. Cell Stem Cell.

