# Supplementary figures and images for: Cell Hierarchy and Lineage Commitment in the Bovine Mammary Gland
Source: PLoS One. 2012 Jan 13;7(1):e30113. doi: 10.1371/journal.pone.0030113 (PMC3258259; doi:10.1371/journal.pone.0030113)

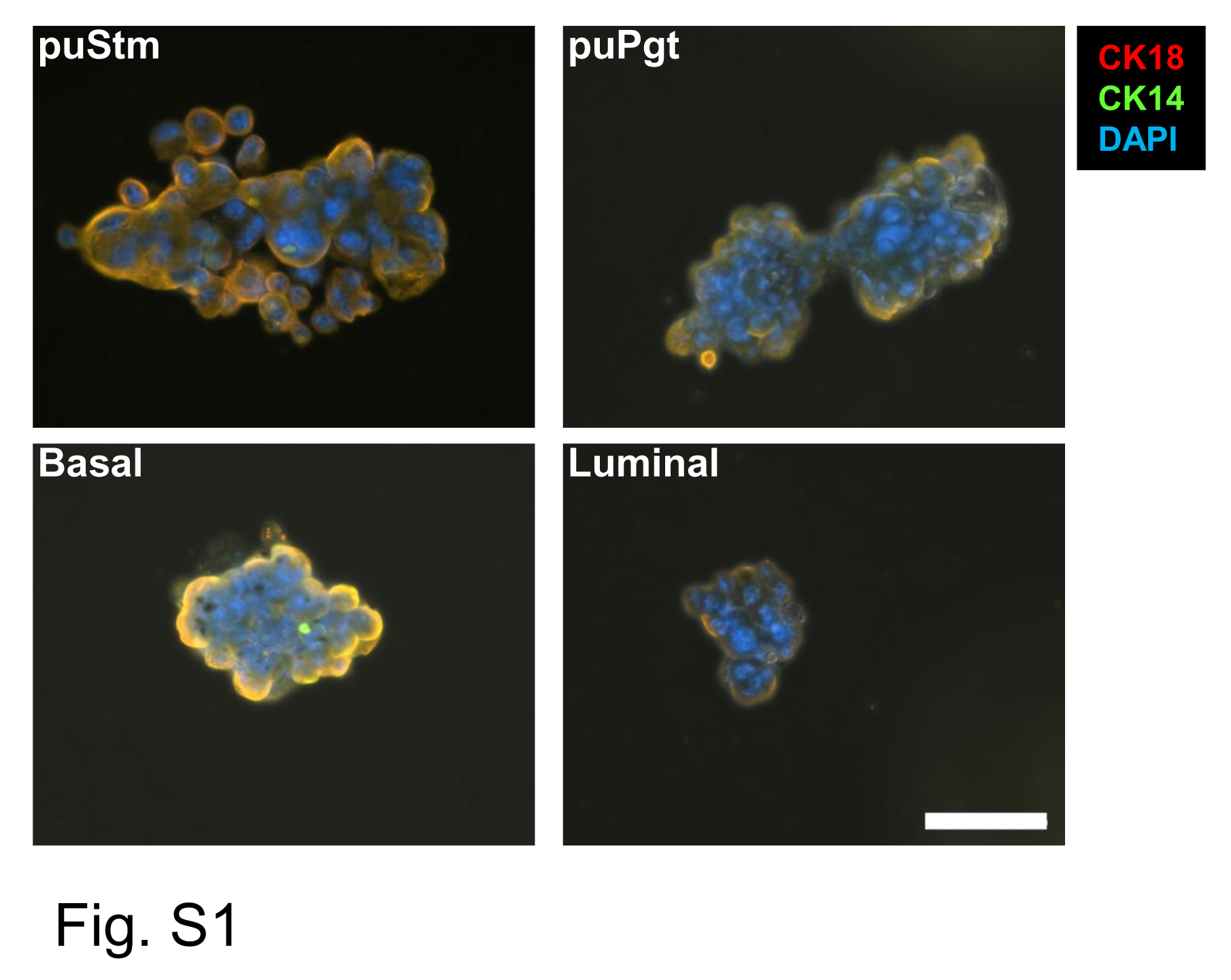

Supplement: Figure S1 — Immunofluorescence staining of single representative NSFCs formed by each sorted population. Bar = 50 µm. (TIF) [file pone.0030113.s001.tif]
