# Supplementary material for: Cell Hierarchy and Lineage Commitment in the Bovine Mammary Gland
Source: PLoS One. 2012 Jan 13;7(1):e30113. doi: 10.1371/journal.pone.0030113 (PMC3258259; doi:10.1371/journal.pone.0030113)
Supplement: Table S1 — List of primers used to amplify coding regions of the listed genes. (DOCX) [file pone.0030113.s002.docx]

| **Gene** | **Gene ID** | **Forward primer (5'→3')** | **Reverse primer (5'→3')** | **Product size (bp)** |
| --- | --- | --- | --- | --- |
| 16S |  | aacatcacctccagcattcc | ttcacgggaaggtcaatttc | 189 |
| HPRT1 | 281229 | TGCCTTGGTCAAGAAGCA | TTCGAGGGGTCCTTTTCA | 70 |
| p63 (TP63) | 615335 | cgaagatccccagatgatg | tgcatgagttccagggatt | 98 |
| CD49f (ITGA6) | 535043 | acccaccggttacaatcct | cttgacgacagcccagatt | 84 |
| CK6 (KRT6A) | 614456 | GGAGCAGATCAAGACCCTCA | CACTTGGTGTCCAGGACCTT | 93 |
| CK14 (KRT14) | 404111 | gcagatcgagagcctcaag | ggcctctcagggagttcat | 77 |
| CK18 (KRT18) | 506480 | atacctaccgccgtctgct | GGATGGTTTGCCTGGAGTT | 84 |
| ERα (ESR1) | 407238 | cgcaagtgctatgaagtgg | cgcttgtgcttcaacattc | 80 |
| GATA3 | 505169 | ccaatggagaccctgtctg | ctggtctggatcccttcct | 97 |
| ALDH1 | 281615 | ccttgcattgtgtttgctg | aacactggccctggtgata | 85 |
| PR | 280895 | GCAGGTCTACCAGCCCTATCTC | GGGCTCTGGCTGGCTTCTGAAT | 60 |
| Nestin | 522383 | AAGGCTTCCCTCAGCTTTC | GGGTCCCAGGGAAATGTAG | 55 |
| Cx32 (GJB1) | 281194 | ACACGCCTGCAGACATTC | AGGTGTACAAACCCGTCCA | 84 |
| Notch1 | 767866 | AACGAGTTCGTGTGCGAGT | GTTCTTGCAGGGTGTGCTT | 90 |
| Delta1 (DLL1) | 788775 | AGGGCCAGTACTGCACAGA | AAAATCCGTGCTGCTCATC | 60 |
| Jagged1 (JAG1) | 783681 | TCCCACTGGTTTCTCTGGA | CTGGCTCGGTTGTAGCACT | 96 |
| Hey1 | 408005 | ACACACTGCAGGAGGGAAA | CCCCAAACTCCGATAGTCC | 67 |
| STAT5A | 282375 | GCAGCCATCTCGAGGACTA | GAACCACTGCCAGAAGGTG | 98 |
| LGR5 | 520189 | AGTTGTTCAGCCTCCGATCT | TGGAAAATGCATTAGGGTCA | 81 |

**Table S1.** List of primers used to amplify coding regions of the listed genes.
